# Expanding Role of Endogenous Biomarkers for Assessment of Transporter Activity in Drug Development: Current Applications and Future Horizon

**DOI:** 10.3390/pharmaceutics16070855

**Published:** 2024-06-25

**Authors:** Vikram Arya, Joseph D. Ma, Kine Eide Kvitne

**Affiliations:** 1 Division of Infectious Disease Pharmacology, Office of Clinical Pharmacology, Center for Drug Evaluation and Research, US Food and Drug Administration, 10903 New Hampshire Avenue, Silver Spring, MD 20993, USA; 2 Skaggs School of Pharmacy & Pharmaceutical Sciences, University of California, San Diego, CA 92093, USA; 3 Department of Pharmacy, University of Oslo, 0316 Oslo, Norway

**Keywords:** drug transporters, drug–drug interactions, endogenous biomarkers, renal impairment, hepatic impairment

## Abstract

The evaluation of transporter-mediated drug–drug interactions (DDIs) during drug development and post-approval contributes to benefit–risk assessment and helps formulate clinical management strategies. The use of endogenous biomarkers, which are substrates of clinically relevant uptake and efflux transporters, to assess the transporter inhibitory potential of a drug has received widespread attention. Endogenous biomarkers, such as coproporphyrin (CP) I and III, have increased mechanistic understanding of complex DDIs. Other endogenous biomarkers are under evaluation, including, but not limited to, sulfated bile acids and 4-pyridoxic acid (PDA). The role of endogenous biomarkers has expanded beyond facilitating assessment of transporter-mediated DDIs and they have also been used to understand alterations in transporter activity in the setting of organ dysfunction and various disease states. We envision that endogenous biomarker-informed approaches will not only help to formulate a prudent and informed DDI assessment strategy but also facilitate quantitative predictions of changes in drug exposures in specific populations.

## 1. Background

Transporters are membrane-bound proteins, which, either alone or in concert with drug metabolizing enzymes, can impact systemic exposure of exogenous and endogenous substances [1]. A thorough evaluation of transporter-mediated drug–drug interactions (DDIs) during drug development and post-approval is important because drugs may interact with transporters as substrates or modulators (inhibitors or inducers). Results from well-conducted DDI trials can help formulate clinical management strategies for the optimal use of drugs in the intended patient population and contribute to the overall benefit–risk assessment [2,3].

Several publications from the International Transporter Consortium (ITC) group have highlighted the progress made in identifying and evaluating the role of clinically relevant transporters, outlined decisional roadmaps that may be used to assess the need for conducting an in vivo DDI trial, and described knowledge gaps for reliable in vitro to in vivo extrapolation (IVIVE) [4,5,6,7,8]. Advancements in transporter research have also significantly contributed to understanding the effect of extrinsic and intrinsic factors on transporter activity and application of quantitative tools to predict the potential of transporter-mediated DDIs, and unraveled the role of endogenous biomarkers to assess transporter activity.

Assessing the inhibitory effect of an investigational drug on various transporters is an integral part of a comprehensive clinical pharmacology program. This assessment entails a combination of in vitro, in silico (for example, mechanistic static or physiologically based pharmacokinetic [PBPK] modeling), and in vivo approaches. Specifically, the potential of a drug to inhibit transporters is predicted by comparing the ratio of the relevant concentration of a drug (for example, for the organic anion transporting polypeptide [OATP]1B1transporter, the hepatic inlet concentration is the most relevant concentration) and the half maximum inhibitory concentration (IC_50_) to a pre-defined “cut-off” value. If the ratio is below the “cut-off” value, no additional assessment of DDIs is recommended, whereas if the ratio is above the “cut-off” value, modeling approaches (a mechanistic static model or a PBPK model) or an in vivo DDI trial may be recommended [9].

Retrospective analyses of the various “cut-off” values used to assess the potential for a drug to inhibit transporters have clearly demonstrated that in some cases, despite the ratio being below a pre-defined “cut-off” value, DDIs were observed (false negative) and despite the ratio being above the “cut-off” value, no significant DDIs were observed (false positive) [10,11]. A variety of factors have been implicated for the false negative and false positive predictions [12]. Such factors include reliably determining the extent of transporters’ contribution to drug uptake and laboratory-to-laboratory variability in the estimate of in vitro inhibitory potency. Hence, there is a motivation to refine the current approaches to assess the potential for a drug to inhibit transporters [13]. 

The use of endogenous biomarkers to assess the potential of a drug to inhibit transporter activity, inform the potential for transporter-mediated DDIs, and guide DDI risk assessment has received widespread attention [14,15,16,17]. Endogenous biomarkers are compounds that are substrates of various uptake and efflux transporters; hence, alteration in endogenous biomarker concentrations, when evaluated in conjunction with other information, may more reliably predict the potential for transporter-mediated DDIs relative to current approaches. Endogenous biomarkers have also helped shed light on alterations in transporter activity in the setting of organ dysfunction and enabled the prediction of DDIs in specific populations such as patients with renal impairment [18,19]. 

The purpose of this article is to highlight how the incorporation of endogenous biomarker assessments as part of the overall DDI assessment strategy can provide mechanistic insights into observed DDIs and enable robust quantitative DDI predictions. Further, the manuscript summarizes how endogenous biomarkers have shed light on alterations in transporter activity due to organ impairment or various disease states. 

## 2. Role of Coproporphyrins in Informing the Potential for Transporter-Mediated DDIs

Coproporphyrin (CP) I and III are promising endogenous biomarkers for assessing the potential for a drug to inhibit OATP1B transporters [20,21]. CPI and CPIII are organic anions and porphyrin metabolites of heme biosynthesis. They are taken up into the hepatocyte by OATP1B1 and OATP1B3 and then eliminated either by urinary elimination or by biliary efflux mediated via the multidrug resistance-associated protein (MRP) 2 and MRP3 transporters [22]. Yee et al. [23] evaluated the impact of the OATP1B1 functional variant, c.521T>C (rs4149056), on the plasma concentrations of CPI and CPIII and showed that unlike CPI, plasma concentrations of CPIII were not sensitive to *SLCO1B1* (solute carrier organic anion transporter family member 1B1) genotypes, suggesting the importance of additional transporters (such as OATP1B3) in the disposition of CPIII. 

The potential role of CPI and CPIII as endogenous biomarkers for assessing OATP1B inhibition was demonstrated by Lai et al., who evaluated the effect of a single dose of rifampin (OATP1B and P-glycoprotein [P-gp]inhibitor) on rosuvastatin (substrate of OATP1B1/OATP1B3, organic anion transporter (OAT)3, and breast cancer resistance protein [BCRP]), CPI, and CPIII [22]. Rifampin increased the maximum concentration (C_max_) of rosuvastatin by approximately 13-fold and the area under the plasma concentration curve (AUC) from time 0 to 24 h (AUC_0–24h_) by 5-fold. When rosuvastatin was administered alone, no changes in CPI and CPIII concentrations were noted. However, after the co-administration of rifampin and rosuvastatin, C_max_ and AUC_0–24h_ of CPI increased by 5.7-fold and 4-fold, respectively, and C_max_ and AUC_0–24h_ of CPIII increased by 6.5-fold and 3.3-fold, respectively. Overall, simultaneous assessment of pharmacokinetic changes in an exogenously administered known substrate of OATP1B such as rosuvastatin and CPI and CPIII sheds light on the potential role of these endogenous biomarkers in assessing DDIs mediated by the inhibition of OATP1B transporters. 

Heinig et al. [24] assessed the in vitro and in vivo inhibitory effect of finerenone on BCRP, OATP1B1, and OATP1B3 transporters. Based on the results of the in vitro assessments, finerenone was expected to inhibit all these transporters in vivo. Further, metabolite M1a was expected to inhibit OATP1B1 and metabolite M3a was expected to inhibit OATP1B1 and OATP1B3. However, the results of the DDI trial did not show any significant changes in the exposure of rosuvastatin. In addition, no alterations in the plasma concentrations of CPI and CPIII were noted, which further illustrates how a strategic incorporation of endogenous biomarkers can inform the transporter inhibitory potential (or lack thereof) of a drug. 

Endogenous biomarkers can also provide mechanistic insights into observed DDIs. Shimizu et al. [25] evaluated the DDI potential of ensitrelvir with substrates of various transporters. When ensitrelvir was co-administered with rosuvastatin as part of a transporter probe cocktail study, the C_max_ and AUC_0–inf_ of rosuvastatin increased by 97% and 65%, respectively. Although it was speculated at the time that the observed changes in rosuvastatin concentrations are primarily due to BCRP inhibition, no definitive conclusions could be drawn regarding the role (or lack thereof) of OATP1B inhibition in the observed DDI. Subsequently, CPI concentrations were analyzed using the plasma samples collected in the study to evaluate the OATP1B inhibitory potential of ensitrelvir [26]. The results showed no significant change in CPI concentrations with/without the co-administration of ensitrelvir, thereby suggesting that the observed DDI was likely due to BCRP inhibition by ensitrelvir. Similarly, based on no observed changes in the concentration of CPI, Piscitelli et al. concluded that an increase in rosuvastatin concentrations after co-administration with encorafenib (an inhibitor of OATP1B and BCRP transporters based on in vitro assessments) can be attributed to the inhibition of BCRP transporters [27]. 

Endogenous biomarkers have also facilitated delineating the involvement of multiple transporters in drug disposition. Mukker et al. [28] evaluated the effect of the intravenous administration of a single dose of rifampin on the pharmacokinetics of orally administered trazpiroben, a substrate of P-gp and OATP1B transporters in vitro [29]. The trial was designed to delineate the inhibitory effect of rifampin towards OATP1B and P-gp transporters. The results of the trial showed an approximately 5-fold increase in AUC_0–inf_ and a 6-fold increase in C_max_ of trazpiroben when co-administered with rifampin vs. when given alone. Further, AUC_0–24h_ and C_max_ of CPI increased by approximately 3.2-fold and 4.9-fold, respectively, and AUC_0–24h_ and C_max_ of CPIII increased by 2.9-fold and 4.3-fold, respectively, thereby suggesting OATP1B inhibition as a mechanistic explanation for the observed DDI. 

Typically, a wide range of inhibitor exposures may be assessed in single- and multiple-dose-ascending studies. Incorporating assessment of endogenous biomarker concentrations in such studies can provide an early read out of the transporter inhibitory potential of a drug. If there are signals of transporter inhibition, as assessed by changes in the concentration of endogenous biomarkers, this information can be leveraged to inform subsequent DDI assessment strategy. Tess et al. [30] assessed the OATP1B1 inhibitory effect of PF-06835919 (a ketohexokinase inhibitor) by measuring changes in CPI plasma concentrations and demonstrated a dose-dependent increase in the concentrations of CPI in the 100-600 mg PF-06835919 dose range in a single-ascending-dose (SAD) trial and after the once-daily administration of PF-06835919 (50 mg and 280 mg). A “middle out” PBPK model for CPI (previously developed by the same authors and refined to account for the lower baseline levels of CPI in control groups of single- and multiple-ascending-dose trials), with in vitro-generated Ki,_OATP1B_, was used to predict the CPI concentrations following single- and multiple-dose administrations of PF-06835919. The model was then used to predict the effect of PF-06835919 on atorvastatin exposures (predicted AUC ratio of ~1.14 and 1.53 at PF-06835919 doses of 50 mg and 280 mg once daily) and the predictions showed good concordance with the observed changes in atorvastatin exposures when co-administered with PF-06835919 (observed AUC ratio of 1.14 and 1.54 at PF-06835919 doses of 50 mg and 280 mg once daily, respectively). Overall, the authors demonstrated how information on CPI early in drug development, in conjunction with mechanistic models, may be strategically leveraged to inform the potential for OATP1B inhibition across a wide range of dosing regimens of the inhibitory drug. 

The incorporation of endogenous biomarker assessment in drug development programs, where appropriate, can guide transporter-mediated DDI assessment strategy and enable robust DDI predictions [14,15]. Kikuchi et al. [13] described work aimed to demonstrate how the assessment and interpretation of changes in CPI concentrations may help to prioritize, delay, or replace DDI trials. The analysis showed that negative results for CPI (defined in the publication as C_max_R < 1.25; C_max_R is the ratio of the maximum concentration of CPI with and without the inhibitor) were associated with “negative” OATP1B substrate DDIs (defined as AUCR < 1.25; AUCR is the ratio of the substrate AUC with and without the inhibitor). The authors suggested that C_max_R < 1.25 is indicative of a lack of significant in vivo OATP1B inhibition. Further, if C_max_R > 1.25, the magnitude of CPI changes was recommended as a guide to inform subsequent decision making. 

Based on the foregoing discussion, the role of coproporphyrins in formulating DDI assessment strategy and providing mechanistic insight into complex DDIs is well recognized. The advantages conferred by an endogenous biomarker-guided DDI assessment approach using coproporphyrins have also fueled interest in exploring the potential utility of other endogenous biomarkers. 

## 3. Evaluation of Endogenous Biomarkers Other Than Coproporphyrins for Assessing Changes in OATP1B Activity

Besides CPI and CPIII, several other endogenous biomarkers have also been reported as potential candidates for assessment of changes in OATP1B activity. Orozco et al. [31] evaluated various sulfated bile acids (BA-S) and determined that glycochenodeoxycholic acid 3-*O*-sulfate (GCDCA-S) and glycodeoxycholic acid 3-*O*-sulfate (GDCA-S) are substrates of OATP1B1, OATP1B3, and sodium taurocholate co-transporting polypeptide (NTCP) transporters with minimal uptake from other SLC transporters such as OATP2B1, OAT2, and OCT1. Further, they assessed the plasma concentrations of GCDCA-S and GDCA-S in *SLCO1B1*-genotyped subjects. Relative to the T/T genotype (reference), mean fasting GDCA-S concentrations were 2.6-fold and 1.3-fold higher in subjects homozygous (C/C genotype) and heterozygous (T/C) for the *SLCO1B1* c.521T>C reduced-function allele, respectively. For GCDCA-S, no significant differences were noted. Overall, the authors concluded that GCDCA-S and GDCA-S are viable endogenous biomarkers of OATP1B but both are less OATP1B1-selective when compared with their corresponding 3-*O*-glucuronides (GCDCA-3G and GDCA-3G). Neuvonen et al. [32] investigated the sensitivity and specificity of GCDCA-3G and GDCA-3G using a comprehensive genomic analysis in healthy volunteers. In vitro, both GCDCA-3G and GDCA-3G showed at least a 6-fold higher uptake by OATP1B1 compared with OATP1B3 or OATP2B1. Further, the mean plasma concentrations of GCDCA-3G and GDCA-3G were 9.2-fold and 6.4-fold higher, respectively, in individuals with the *SLCO1B1* c.521 C/C genotype than in those with the c.521T/T genotype. Overall, the authors noted that GCDCA-3G is a highly sensitive and specific OATP1B1 endogenous biomarker in humans. Neuvonen et al. [33] recently evaluated the performance of CPI, CPIII, GCDCA-3G, and GDCA-3G to assess OATP1B1 activity in a Finnish cohort with various *SLCO1B1* genotypes. Both CPI and GCDCA-3G were considered useful biomarkers to assess OATP1B1 activity and the authors concluded that GCDCA-3G was more sensitive than CPI in assessing changes in OATP1B1 activity. 

Chan et al. [34] evaluated the selectivity of several endogenous biomarkers of OATP1B using a newly developed relative activity factor (RAF) method and determined the contribution of hepatic uptake transporters for several OATP1B biomarkers such as CPI, CPIII, GCDCA-sulfate (S), GDCA-S, and taurochenodeoxycholic sulfate (TCDCA-S). The authors showed that CPI is a more selective endogenous biomarker for OATP1B1 than CPIII (fraction transported by OATP1B1 was 0.57 and 0.21 for CPI and CPIII, respectively). OATP1B3 was shown to be the major contributor for hepatic uptake of GCDCA-S and TCDCA-S while OATP1B1 and OATP1B3 equally contributed to the hepatic uptake of GDCA-S. 

## 4. Evaluation of Endogenous Biomarkers for Assessment of Other (Non-OATP1B) Transporters

Although most of the efforts so far have focused on evaluating endogenous biomarkers for assessing the potential for a drug to inhibit OATP1B transporters, the role of endogenous biomarkers to assess alteration in the activity of other transporters has also been explored.

Changes in the plasma concentrations of isobutyryl-l-carnitine (IBC), a metabolite of valine, have been shown to reflect the inhibition of hepatic organic cation transporter (OCT) 1 [35]. Because OCT1 primarily mediates the efflux of IBC out of the liver, plasma levels of IBC decrease upon OCT1 inhibition. The potential for IBC to inform changes in OCT1 activity was also shown by Jensen et al., who found that individuals carrying the *SLC22A1* genotype associated with normal activity of OCT1 had approximately 3-fold higher plasma levels of IBC than individuals with the *SLC22A1* genotype associated with low activity [36]. Similarly, Matthaei et al. found up to a 5-fold difference in IBC concentrations depending on the *SLC22A1* genotype [37]. 

Shen et al. [38] demonstrated that plasma 4-pyridoxic acid (PDA) levels can be a promising endogenous biomarker for assessing the activity of renal organic anion transporters (OATs) 1 and 3. Relative to baseline, the magnitude of the increase in the plasma concentrations of PDA after the administration of probenecid (an inhibitor of OAT1 and OAT3) was similar to the magnitude of the increase in plasma concentrations of PDA when probenecid was co-administered with furosemide (an exogenously administered probe substrate of OAT1 and OAT3) relative to when furosemide was given alone. Further, after the administration of probenecid, the increase in PDA AUC was similar to the increase in furosemide AUC, thereby suggesting suitability of PDA as an endogenous biomarker for OAT1/3 inhibition. 

The potential role of kynurenic acid as an endogenous biomarker to inform the potential for a drug to inhibit OAT1 and OAT3 transporters has also been investigated [39]. In a DDI trial conducted to assess the effect of probenecid on furosemide pharmacokinetics, plasma concentrations of several endogenous biomarkers (including kynurenic acid) were measured. Relative to baseline, the C_max_ of kynurenic acid was increased by 3.3-fold and 3.8-fold, after the administration of probenecid alone and co-administration of probenecid and furosemide, respectively. Relative to furosemide alone, AUC_0–24h_ of kynurenic acid increased by 2.1-fold when probenecid was administered alone and 2.4-fold when probenecid was co-administered with furosemide. Of note, probenecid increased the C_max_ and AUC_0–inf_ of furosemide by 1.8-fold and 3.3-fold, respectively, and based on the similarity of changes in kynurenic acid and furosemide concentrations, the authors postulated that kynurenic acid can be a potential endogenous biomarker for OAT1/3 inhibition. 

Taurine and GCDCA-S have also been suggested as potential endogenous biomarkers for OAT1 and OAT3, respectively [40]. The DDI sensitivity of these endogenous biomarkers and PDA was investigated by Willemin et al. in healthy volunteers receiving probenecid [41] Although both PDA and GCDCA-S were considered sensitive to OAT inhibition, plasma levels of PDA were considered to be the most informative to detect OAT1 and OAT3 inhibition. GCDCA-S is selective towards OAT3 and measurement of this biomarker together with PDA can therefore be used to deconvolute inhibition by OAT1 and OAT3. 

*N*^1^-methylnicotinamide (NMN), an endogenous metabolite formed from nicotinamide, was shown to be an endogenous biomarker for multidrug and toxin extrusion (MATE) proteins 1/2K and OCT 2 transporters in the kidney [42,43,44,45]. Wang et al. [46] evaluated the potential inhibitory effect of ritlecitinib and its major inactive metabolite M2 on hepatic OCT1 and renal transporter OCT2 and MATE 1/2K using sumatriptan (OCT1 probe drug) and IBC and NMN as endogenous biomarkers for OCT1 and OCT2/MATE 1/2K, respectively. After the co-administration of ritlecitinib and sumatriptan, an increase in sumatriptan AUC_0–inf_ by approximately 30% was observed, which suggests that ritlecitinib and M2 are inhibitors of OCT1. AUC_0–24h_ of IBC was decreased by approximately 15% (lower levels of IBC are associated with decreased activity of OCT1), which is consistent with OCT1 inhibition. The AUC_24h_ and C_max_ of NMN decreased by approximately 20% and 10%, respectively, thereby suggesting a non-significant impact of ritlecitinib on OCT2 and MATE-1/2K transporters.

## 5. Incorporating Assessment of Endogenous Biomarkers in Exogenously Administered Probe Cocktail Trials

A probe “cocktail” approach enables exploring the inhibitory/induction effect of a drug on the pharmacokinetics of several simultaneously administered enzyme and transporter probe drugs [47,48,49]. A well-designed probe cocktail trial can reduce the number of standalone DDI trials and help to efficiently address the pertinent DDI-related questions.

Vourvahis et al. [50] demonstrated how the integration of endogenous biomarker assessment in an exogenously administered probe cocktail trial provides useful information regarding modulation (or lack thereof) in the activity of various transporters. The authors assessed the inhibitory effect of abrocitinib on various transporters by assessing the pharmacokinetics of dabigatran (after administration of dabigatran etexilate; a P-gp substrate), rosuvastatin (substrate of BCRP and OAT3), metformin (substrate of OCT2 and MATE1/2K), NMN, and IBC. Although abrocitinib was shown to inhibit P-gp, it did not show a significant effect on the pharmacokinetics of metformin, rosuvastatin, NMN, and IBC. Lack of significant changes in the concentrations of the aforementioned probe drugs and endogenous biomarkers indicated that abrocitinib did not inhibit MATE1/2K, OCT1, and OCT2 transporters. Additionally, the availability of pharmacokinetic data of the exogenously administered probes and endogenous biomarkers in the same trial contributes to the growing body of evidence related to the use of NMN and IBC as potential endogenous biomarkers to assess the inhibitory potential of a drug towards MATE1/2K and OCT1 transporters, respectively.

Huh et al. [51] evaluated the impact of ritlecitinib using a combination of an exogenous probe drug (rosuvastatin) and endogenous biomarkers (CPI and PDA; assessed to deconvolve any potential changes in rosuvastatin exposures). Based on the results of in vitro studies, ritlecitinib has the potential to inhibit BCRP, OATP1B1, and OAT3 transporters. In a DDI trial subsequently conducted, ritlecitinib decreased rosuvastatin C_max_ and AUC_inf_ by 27% and 13%, respectively. The renal clearance of rosuvastatin and the AUC_inf_ and C_max_ of CPI and PDA were similar after the administration of rosuvastatin alone and after the co-administration of ritlecitinib and rosuvastatin, thereby suggesting that ritlecitinib is not an inhibitor of BCRP, OATP1B1, and OAT3. Interestingly, the authors underscored the importance of measuring endogenous biomarkers in the trial by indicating that had there been a significant increase change in rosuvastatin exposures, availability of information regarding changes in CPI and PDA concentrations would help to explain which transporter is involved in the DDI.

## 6. Expanding the Role of Endogenous Biomarkers beyond Assessment of Transporter-Mediated DDI in Healthy Subjects

Interindividual variability in the activity of drug transporters due to various extrinsic (diet, DDIs) and intrinsic (age, genetics, disease state) factors can impact the safety and efficacy profile of drugs. Hence, understanding the mechanisms underpinning variability in transporter activity is critical to predict individual drug response. There is a growing interest in exploring the application of endogenous biomarkers to assess changes in transporter activity during a disease state. 

Patients with renal impairment have been shown to have higher concentrations of CPI and exogenous probe drug pitavastatin relative to subjects with normal renal function [52]. Interestingly, in the presence of rifampin, the AUC ratio of CPI, but not pitavastatin, was higher in patients with severe renal impairment relative to the AUC ratio in healthy subjects. To investigate mechanisms leading to higher levels of CPI in patients with severe renal impairment relative to healthy subjects and the inconsistent magnitude of OATP1B-mediated increases in CPI and pitavastatin in patients with chronic kidney disease (CKD), Takita et al. [18] used PBPK modeling based on multiple CKD-related covariates. The analysis indicated that patients with CKD have a disease-related decrease in OATP1B activity, leading to higher concentrations of CPI and pitavastatin. Further, due to a decrease in the renal elimination of CPI in CKD, there is a shift in the fraction of CPI eliminated by OATP1B, thereby resulting in higher exposures of CPI upon the administration of rifampin. In contrast, such a fractional shift was not expected for pitavastatin because it only undergoes hepatic elimination. This illustrates how the integration of an endogenous probe such as CPI can help to predict the DDI of drugs with combined hepatic and renal elimination, in patients with CKD. 

Lin et al. [19] recently investigated plasma levels of CPI in subjects with various degrees of hepatic impairment (n = 24) and showed that relative to healthy controls, the geometric mean exposure of CPI was increased by approximately 1.7-, 2.8-, and 7.8-fold in subjects with mild, moderate, and severe hepatic impairment, respectively. A dataset of 21 OATP1B substrate drugs was used to analyze changes in AUC in subjects with varying degrees of hepatic impairment versus healthy controls and the analysis showed a median AUC ratio of 1.4, 3, and 6.4 in subjects with mild, moderate, and severe hepatic impairment, respectively. Furthermore, a linear relationship was identified between the AUC ratios with/without hepatic impairment (moderate and severe) and AUC ratios with/without the co-administration of OATP1B substrates with rifampin. A previously developed PBPK model of CPI was used to predict changes in CPI concentration in subjects with varying degrees of hepatic impairment and compared with the observed CPI concentrations from the aforementioned hepatic impairment trial. Subsequently, PBPK modeling of 10 OATP1B substrate drugs was conducted to predict changes in PK due to hepatic impairment. The authors indicated that the model adequately predicted changes in AUC, particularly in subjects with moderate or severe hepatic impairment. Overall, the authors demonstrated how an endogenous biomarker-guided approach can be used to estimate reduction in transporter activity and facilitate the prediction of dosing recommendations in patients with varying degrees of hepatic impairment. 

Endogenous biomarkers can facilitate assessment of transporter-mediated DDI in patients, in cases where conducting DDI trials in healthy volunteers may pose challenges. This was shown by Mori et al. [53], who investigated the OATP1B1/3 inhibition potency of paclitaxel in therapeutic doses in patients with non-small cell lung cancer by assessing changes in the plasma concentrations of multiple endogenous biomarkers of OATP1B1/3, including CPI, CPIII, sulfate-conjugated bile acids, and glucuronide-conjugated bile acids. After a 3 h infusion with a therapeutic dose of paclitaxel (200 mg/m^2^), AUC_-3–7h_ (area under the curve from the start of the 3 h infusion to the last PK sampling time [samples were collected at 0, 2, 4, and 7 h after the end of the infusion]) of the endogenous biomarkers increased by 2- to 4-fold, suggesting that paclitaxel inhibits OATP1B transporters. This illustrates how endogenous biomarkers may be used to inform the potential for transporter-mediated DDIs in the target patient population. 

## 7. Quantification and Metabolomics-Informed Identification of Endogenous Biomarkers

The accurate quantification of endogenous biomarkers in human plasma and urine is critical to support their role in the assessment of transporter-mediated DDIs. The development, validation, and application of bioanalytical assays used for the quantification of endogenous biomarkers have been documented in detail in the literature [54,55,56]. Kader et al. [57] recently described the development and validation of endogenous biomarker multiplex assays, which can be used to measure concentrations of various endogenous biomarkers and the application of these assays in facilitating transporter-mediated DDI assessment. Metabolomics, which entails the comprehensive analysis of metabolites, has greatly enhanced our ability to identify novel endogenous metabolic biomarkers and multiple studies have shown the value of using metabolomics to identify potential endogenous biomarkers for assessment of transporter activity [58,59,60]. Recently, Jin et al. [61] established stably transfected cells that overexpressed OATP1B1, OATP1B3, and P-gp transporters and highlighted the importance of using metabolomics to identify appropriate endogenous biomarkers. The authors demonstrated that azelaic acid may be a potential biomarker to assess OATP1B3-P-gp function in the liver and underscored the need for further assessments. In part of another study by Zhang et al. [62], a combination of untargeted metabolomics and transporter knockout mice models were used to assess association between BCRP deficiency and circulating levels of endogenous metabolites reflecting BCRP function. Of 243 examined metabolites, riboflavin was identified as a specific BCRP substrate. 

## 8. Summary and Future Directions

The strategic integration of endogenous biomarker assessment in drug development programs, where appropriate, can provide helpful mechanistic insights into observed DDIs and delineate the role of multiple transporters. Although the role of coproporphyrins in facilitating assessment of transporter-mediated DDIs has been extensively evaluated, other endogenous biomarkers such as sulfated bile acids (e.g., GCDCA-S and GDCA-S), PDA, and NMN have also been incorporated to provide useful insights regarding the alteration in activity of OATP1B and various renal transporters. Recently, the suitability of riboflavin as an endogenous biomarker for assessing changes in BCRP transporters has been explored [62,63]. Availability of robust bioanalytical assays to enable assessment of multiple endogenous biomarkers will enable the prioritization of subsequent DDI strategy. The role of endogenous biomarkers has considerably expanded beyond facilitating assessment of transporter-mediated DDIs. Endogenous biomarkers are being used to characterize changes in transporter activity due to hepatic impairment and renal impairment. It is envisioned that assessment of endogenous biomarkers in various sub-populations such as pediatrics, pregnant individuals, and geriatrics will likely play an important role to assess alterations in transporter activity due to physiological changes. 

## Data Availability

Data are contained within the article.
